# Association of serum leptin levels with central arterial stiffness in coronary artery disease patients

**DOI:** 10.1186/s12872-016-0268-5

**Published:** 2016-05-05

**Authors:** Jen-Pi Tsai, Ji-Hung Wang, Mei-Ling Chen, Chiu-Fen Yang, Yu-Chih Chen, Bang-Gee Hsu

**Affiliations:** Division of Nephrology, Department of Internal Medicine, Dalin Tzu Chi Hospital, Buddhist Tzu Chi Medical Foundation, Chiayi, Taiwan; School of Medicine, Tzu Chi University, Hualien, Taiwan; Division of Cardiology, Buddhist Tzu Chi General Hospital, Hualien, Taiwan; Division of Nephrology, Buddhist Tzu Chi General Hospital, Hualien, Taiwan

**Keywords:** Arterial stiffness, Carotid-femoral pulse wave velocity, Coronary artery disease, Leptin

## Abstract

**Background:**

Serum adipokines have roles in the development of arterial stiffness. Our aim was to investigate the relationship of leptin and the surrogate marker carotid-femoral pulse wave velocity (cfPWV) in coronary artery disease (CAD) patients.

**Methods:**

Fasting blood samples were obtained from 105 CAD patients. cfPWV was measured with the SphygmoCor system. A cfPWV > 10 m/s was defined as high arterial stiffness, and ≤ 10 m/s as low arterial stiffness.

**Results:**

Thirty-seven patients (35.2 %) had high arterial stiffness, and had a higher percentage of diabetes (*P* = 0.001), hypertension (*P* = 0.010), older age (*P* = 0.001), and higher systolic blood pressure (SBP) (*P* < 0.001), diastolic blood pressure (DBP) (*P* = 0.021), pulse pressure (*P* = 0.014), and serum leptin level (*P* = 0.002) compared to patients with low arterial stiffness. Serum leptin levels correlated with the number of angiographically documented stenotic coronary artery vessels (*P* < 0.001). After adjusting for factors significantly associated with arterial stiffness, multivariate logistic regression analysis showed that leptin (odds ratio = 1.026, 95 % confidence interval: 1.002–1.051, *P* = 0.037) was a significant independent predictor of arterial stiffness.

**Conclusions:**

Increasing serum concentration of leptin correlated positively with the total number of stenotic coronary arteries, and serum leptin level may predict the development of arterial stiffness in CAD patients.

## Background

Arterial stiffness is one of the multiple characteristics of cardiovascular disease (CVD), and the mechanisms of arterial stiffness most often include abnormal regulation of elastin fibers and collagen, re-organization of cellular elements, and low-grade inflammation [1–3]. One meta-analysis showed increased pooled relative risks for total CV events, CV mortality, and all-cause mortality for subjects with high versus low brachial-ankle pulse wave velocity (PWV) [4]. PWV has been recognized as a non-invasive method to access vascular function, and as the gold standard surrogate marker of arterial wall function and structure [5]. A systematic review showed that aortic PWV is a strong predictor of future CV events and all-cause mortality, and has a predictive value independent of classical CV risk factors [6].

Studies have shown a relationship between CVD and adipokines such as leptin, adiponectin, and resistin [7, 8]. Leptin was discovered in 1994, and is a 16-kDa product of the obese gene mainly produced by white adipose tissue, and is involved in a wide range of functions beyond fat storage [9]. Classical effects of leptin include food intake reduction and increased energy expenditure, and its levels are directly associated with white adipose tissue [10]. Hyperleptinemia has been implicated in metabolic, inflammatory, and homeostatic factors involved in obesity, hypertension (HTN), and CVD [11–13]. Leptin has been associated with enhanced neointimal and medial thickening of injured carotid artery vascular walls, and stimulates the renin-angiotensin-aldosterone system, the proliferation of vascular smooth muscle cells, endothelial oxidative stress, and the formation of reactive oxygen species, which contributed to the development of aortic mechanical dysfunction and arterial stiffness [12, 14–16]. Increased PWV had been shown to predict a greater risk of CV morbidity and mortality in hypertensive and diabetic patients, but the relationship of serum leptin and arterial stiffness in patients with coronary artery disease (CAD) is not clear. Thus, the aim of this study was to examine the risk factors contributing to arterial stiffness measured by PWV in angiographically diagnosed CAD patients, to examine the correlation of serum leptin and carotid-femoral PWV, and to observe the relationship between serum leptin and severity of CAD (number of stenotic coronary arteries).

## Methods

### Patients

The Protection of the Human Subjects Institutional Review Board of Tzu-Chi University and Hospital approved this study. Between March and December 2012, 105 CAD patients (79 males and 26 females; CAD defined as > 50 % stenosis in any segment by coronary angiography) in a medical center in Hualien, eastern Taiwan were enrolled in this study. Trained staff measured blood pressure (BP) in the morning for all participants using a standard mercury sphygmomanometer with appropriate cuff size after the patients had been sitting for at least 10 min. Systolic BP (SBP) and diastolic BP (DBP) were taken at the points of appearance and disappearance, respectively, of the Korotkoff sounds. SBP and DBP were taken three times at 5 min intervals, and were averaged for analysis. Pulse pressure was calculated by subtracting DBP from SBP. HTN was defined as SBP ≥ 140 mmHg, and/or DBP ≥ 90 mmHg, or prescription of antihypertensive medication in the past 2 weeks. A person was regarded as having diabetes mellitus (DM) if the fasting plasma glucose was either 126 mg/dl or more, or if he/she was using diabetes medications (oral or insulin) [17]. Participants were excluded if they had an acute infection, acute myocardial infarction, pulmonary edema at the time of blood sampling, were taking calcium, active vitamin D metabolites, bisphosphonates, teriparatide, or estrogens, or if they declined to provide informed consent for the study.

### Anthropometric analysis

Body weight was measured in light clothing and without shoes to the nearest 0.5 kg, and body height was measured to the nearest 0.5 cm. Body mass index (BMI) was calculated as the weight in kilograms divided by the height in meters squared [18, 19].

### Biochemical investigations

Fasting blood samples (approximately 5 ml) were immediately centrifuged at 3000 *g* for 10 min. Serum levels of blood urea nitrogen (BUN), creatinine (Cre), fasting glucose, total cholesterol (TCH), triglycerides (TG), high-density lipoprotein cholesterol (HDL-C), low-density lipoprotein cholesterol (LDL-C), total calcium, and phosphorus were measured using an autoanalyzer (COBAS Integra 800, Roche Diagnostics, Basel, Switzerland). [18, 19] Serum leptin concentrations were determined using a commercially available enzyme immunoassay (EIA) (SPI-BIO, Montigny le Bretonneux, France) [20]. Calculation of estimate glomerular filtration rate (GFR) was based on the Modification of Diet in Renal Disease (MDRD) equation.

### Carotid-femoral PWV (cfPWV) measurements

Measurement of cfPWV was performed using a pressure tonometer to transcutaneously record the pressure pulse waveform in the underlying artery (SphygmoCor system, AtCor Medical, Australia), as previously described [18, 19]. All measurements were performed in the morning in the supine position after a minimum 10 min rest in a quiet, temperature-controlled room. Recording were made simultaneously with an ECG signal, which provided an *R*-timing reference. Pulse wave recordings were performed consecutively at two superficial artery sites (carotid-femoral segment). Integral software was used to process each set of pulse wave and ECG data to calculate the mean time difference between *R*-wave and pulse wave on a beat-to-beat basis, with an average of 10 consecutive cardiac cycles. The cfPWV was calculated using the distance and mean time difference between the two recorded points. Quality indices, included in the software, were set to ensure uniformity of data. A cfPWV > 10 m/s was defined as high arterial stiffness, and ≤ 10 m/s as low arterial stiffness according to the European Society of Hypertension and the European Society of Cardiology (ESH-ESC) 2013 Guidelines [5].

### Statistical analysis

Normally distributed data were expressed as mean ± standard deviation (SD), and comparisons were performed using the Student’s independent t-test (two-tailed). Data not normally distributed were expressed as medians and interquartile ranges, and comparisons were performed using the Mann–Whitney *U* test (TG, fasting glucose, BUN, Cre, and leptin). Data expressed as the number of patients were analyzed by the *χ*^2^ test. Differences of leptin levels between numbers of occluded vessels was analyzed by the Kruskal-Wallis analysis of variance (AVONA) test. Variables that were significantly associated with arterial stiffness were tested for independence by multivariate logistic regression analysis (adapted factors: DM, HTN, age, SBP, DBP, pulse pressure, and leptin). Data were analyzed using SPSS for Windows (version 19.0; SPSS Inc., Chicago, IL, USA). Values of *P* < 0.05 were considered statistically significant.

## Results

Demographic, biochemical, and clinical characteristics of the 105 CAD patients are shown in Tables 1 and 2. A total of 51 patients (48.6 %) had DM and 53 (50.5 %) HTN. The use of drugs included angiotensin receptor blockers (ARB; *n* = 28; 26.7 %), angiotensin-converting enzyme inhibitors (ACEi; *n* = 23; 21.9 %), calcium channel blockers (CCB; *n* = 35; 33.3 %), β-blockers (*n* = 49; 46.7 %), statins (*n* = 69; 65.7 %), and fibrate (*n* = 21; 20.0 %). Thirty-seven patients (35.2 %) were defined as high arterial stiffness, and this group of patients had a higher percentage of DM (*P* = 0.001) and HTN (*P* = 0.010) as compared to the low arterial stiffness group. There was no statistically significant difference in sex and use of ACEi, ARB, β-blockers, CCB, statins, or fibrate between the two groups. Age (*P* = 0.001), SBP (*P* < 0.001), DBP (*P* = 0.021), pulse pressure (*P* = 0.014), and serum leptin level (*P* = 0.002) were higher in the high arterial stiffness group compared with the low arterial stiffness group.

**Table 1:** Clinical variables of the 105 coronary artery disease patients

Characteristic	All participants (*n* = 105)	Low Arterial Stiffness (*n* = 68)	High Arterial Stiffness (*n* = 37)	*P*
Age (years)^a^	65.55 ± 9.19	63.31 ± 8.92	69.68 ± 8.19	0.001*
Height (cm)^a^	161.03 ± 8.19	161.59 ± 7.44	160.00 ± 9.43	0.345
Body weight (kg)^a^	67.80 ± 11.78	67.79 ± 12.39	67.80 ± 10.75	0.998
Body mass index (kg/m^2^)^a^	26.05 ± 3.39	25.82 ± 3.39	26.47 ± 3.40	0.357
cfPWV (m/s)^a^	9.37 ± 2.71	7.81 ± 1.32	12.25 ± 2.20	<0.001*
Systolic blood pressure (mmHg)^a^	132.10 ± 18.67	127.47 ± 16.18	140.59 ± 20.13	<0.001*
Diastolic blood pressure (mmHg)^a^	72.56 ± 10.32	70.85 ± 10.07	75.70 ± 10.16	0.021*
Pulse pressure (mmHg)^a^	59.53 ± 16.64	56.62 ± 14.22	64.89 ± 19.43	0.014*
Total cholesterol (mg/dl)^a^	165.98 ± 35.80	170.15 ± 37.45	158.32 ± 31.59	0.106
Triglyceride (mg/dl)^b^	117.00 (89.50–162.00)	116.00 (90.75–184.50)	117.00 (89.00–152.00)	0.351
HDL-C (mg/dl)^a^	45.33 ± 12.24	46.54 ± 12.15	43.11 ± 12.28	0.171
LDL-C (mg/dl)^a^	95.73 ± 26.58	97.37 ± 26.47	92.73 ± 26.87	0.396
Fasting glucose (mg/dl)^b^	111.00 (96.50–142.50)	107.00 (97.00–133.75)	114.00 (95.50–163.00)	0.509
Blood urea nitrogen (mg/dl)^b^	16.00 (13.00–19.00)	15.50 (13.00–18.00)	16.00 (13.00–21.50)	0.162
Creatinine (mg/dl)^b^	1.10 (0.90–1.30)	1.00 (0.90–1.20)	1.20 (0.90–1.40)	0.143
Glomerular filtration rate (ml/min)^a^	70.55 ± 20.36	73.10 ± 17.63	65.92 ± 24.13	0.085
Total calcium (mg/dl)^a^	9.13 ± 0.36	9.15 ± 0.36	9.08 ± 0.35	0.328
Phosphorus (mg/dl)^a^	3.52 ± 0.53	3.53 ± 0.55	3.49 ± 0.48	0.739
Ca × P product (mg^2^/dL^2^)^a^	32.12 ± 5.15	32.32 ± 5.39	31.74 ± 4.73	0.585
Leptin (ng/ml)^b^	6.58 (3.41–18.97)	5.50 (2.80–13.77)	13.60 (5.07–52.43)	0.002*

Data are expressed as mean ± standard deviation, except for triglycerides, fasting glucose, blood urea nitrogen, creatinine, C-reactive protein, and leptin, which are expressed as median and interquartile range (IQR)

*Abbreviations*: *cfPWV* carotid-femoral pulse wave velocity, Ca × P product, calcium-phosphorus product, *HDL-C*, High density lipoprotein cholesterol, *LDL-C* Low density lipoprotein cholesterol, *iPTH* Intact parathyroid hormone

^a^Data were tested using Student’s t-test

^b^Data were testing using Mann-Whitney *U* test

*P < 0.05 was considered statistically significant by Student’s t-test or Mann-Whitney *U* test

**Table 2:** Baseline characteristics of the 105 coronary artery disease patients

Characteristic		Low Arterial Stiffness	High Arterial Stiffness	*P*
Gender	Male	51 (75.0)	28 (75.7)	0.939
	Female	17 (25.0)	9 (24.3)	
Diabetes mellitus	No	43 (63.2)	11 (29.7)	0.001*
	Yes	25 (36.8)	26 (70.3)	
Hypertension	No	40 (58.8)	12 (32.4)	0.010*
	Yes	28 (41.2)	25 (67.6)	
Angiotensin-converting enzyme inhibitor	No	53 (77.9)	29 (78.4)	0.959
	Yes	15 (22.1)	8 (21.6)	
Angiotensin-receptor blocker	No	50 (73.5)	27 (73.0)	0.951
	Yes	18 (26.5)	10 (27.0)	
β-blocker	No	36 (52.9)	20 (54.1)	0.913
	Yes	32 (47.1)	17 (45.9)	
Calcium-channel blocker	No	48 (70.6)	22 (59.5)	0.248
	Yes	20 (29.4)	15 (40.5)	
Statin	No	22 (32.4)	14 (37.8)	0.572
	Yes	46 (67.6)	23 (62.2)	
Fibrate	No	50 (73.5)	30 (81.1)	0.385
	Yes	14 (26.5)	7 (18.9)	

Data are expressed as number (percentage), and were analysed with the chi-square test

*P < 0.05 was considered statistically significant

**Fig. 1:**
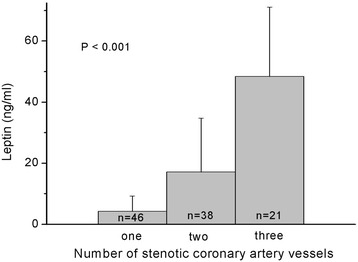
Fasting leptin levels in patients with different numbers of stenotic coronary vessels. Data were analysed by the Kruskal-Wallis analysis of variance (AVONA) test

**Fig. 2:**
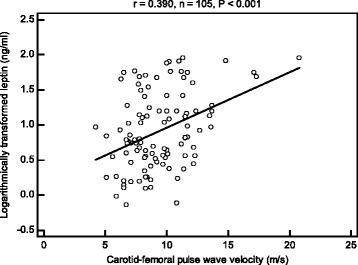
Two-dimensional scatter-plots of carotid-femoral pulse wave velocity and logarithmically transformed leptin levels among the 105 coronary artery disease patients

Fasting serum leptin levels based on the number of stenotic coronary artery vessels are shown in Fig. 1. There was a statistically significant difference between the number of stenotic coronary artery vessels and serum leptin levels (*P* < 0.001). Figure 2 showed two-dimensional scattered plots of logarithmically transformed leptin levels and cfPWV values among the 105 patients and revealed significantly positive correlation (*r* = 0.390; *P* < 0.001).

Multivariate logistic regression analysis of the factors significantly associated with arterial stiffness (age, DM, HTN, SBP, DBP, pulse pressure, and leptin) showed that leptin (*P* = 0.037), age (*P* = 0.002), and DM (*P* = 0.023) were independent predictors of arterial stiffness (Table 3).

**Table 3:** Multivariate logistic regression analysis of factors correlated with arterial stiffness

Variables	Odds Ratio	95 % Confidence Interval	*P*
Leptin (ng/ml), per 1 ng/ml increase	1.026	1.002–1.051	0.037*
Age (years), per 1 year increase	1.105	1.036–1.179	0.002*
Diabetes mellitus, presence	3.369	1.185–9.576	0.023*

*P < 0.05 was considered statistically significant in the multivariate logistic regression analysis (adapted factors: diabetes mellitus, hypertension, age, systolic blood pressure, diastolic blood pressure, pulse pressure, and leptin)

## Discussion

The results of this study showed that fasting leptin level was higher in CAD patients with high arterial stiffness than those with low arterial stiffness, and was an independent predictor for the development of arterial stiffness. In addition, serum concentration of leptin correlated positively with the number of stenotic coronary arteries.

Classic CV risk factors, including DM, hyperlipidemia, elevated BMI, and smoking, have been implicated in accelerating arterial stiffness. Aortic stiffness can affect aortic function and reduce baroreceptor responsiveness, and arterial stiffness one of the leading causes of increased blood pressure [3, 21]. The pathophysiological correlation between arterial stiffness and HTN is that increased arterial stiffness reduces the lumen diameter and leads to a premature return of the reflected wave in late systole, resulting in increased pulse pressure and SBP, and decreased DBP [22]. One systematic reviewed has found that in approximately 90 % of studies, the prognostic value of cfPWV may be independently related to a process of arterial ageing and elevation of BP, and is probably unrelated to classical risk factors such as sex, TCH, LDL-C, HDL-C, triglycerides, smoking, and BMI [23]. Additionally, a population-based study showed that after adjusting for mean arterial pressure, age, and gender impaired glucose tolerance and DM were independently associated with central arterial stiffness [24]. Similarly, we found that CAD patients with high arterial stiffness were older and had higher pulse pressure, SBP, DBP, and a higher percentage had DM and HTN. Furthermore, older age and DM were possible risk factors for the development of arterial stiffness after adjusting covariates.

Leptin is a peptide product of the obese gene, and is predominantly secreted by white adipose tissue [10, 25]. In addition to have a physiological role of regulating hunger and satiety, studies have shown an association between leptin and CVD [7, 8]. Possible roles of leptin with respect to the development of vascular lesions include stimulating phosphorylation and activation of mitogen-activated protein kinases and phosphatidylinositol-3 kinase to increase the proliferation and migration of vascular smooth muscle cells, inducing endothelial dysfunction and enhancing the effects of angiotensin II on BP via modulating the sympathetic nervous system, and inducing formation of reactive oxygen species by increasing fatty acid oxidation via protein kinase A activation in endothelial cells to contribute to the development of arterial stiffness [14, 15, 26, 27]. Several cross-sectional studies which included aged, resistant HTN, and healthy individuals showed that hyperleptinemia was inversely associated with vasodilatation in resistance arteries, and positively with PWV, and hence arterial stiffness [28–30]. These studies, and our previous study conducted in kidney transplantation recipients, revealed a relationship between hyperleptinemia and arterial stiffness measured by brachial-ankle PWV, and indicated that leptin could have a role in the relationship between abdominal adiposity and arterial stiffness, and have an impact on the pathophysiology of macrovascular diseases [28–31]. Moreover, in the current study we found that hyperleptinemia correlated with cfPWV and was an independent risk factor for the development of arterial stiffness in patients with angiographically documented CAD.

In the current study, serum leptin correlated with the total number of diseased coronary arteries. This finding is similar with those of previous studies which revealed that serum leptin plays an important role in the occurrence, severity, and extent of CAD [32–34]. Patients with acute myocardial infarction have been reported to have a trend for an increased serum leptin level with an increasing number of diseased vessels [34]. In patients with angiographically diagnosed coronary atherosclerosis, hyperleptinemia correlated positively with the degree of vessel narrowing, the proportion abnormal coronary artery segments, and the complexity of the atherosclerotic lesions [32]. In diabetic patients, hyperleptinemia was found to be a risk factor for the development of CAD with an area under curve (AUC) of 0.62 (95 % confidence interval [CI]: 0.54-0.7) in men and 0.71 (95 % CI: = 0.6-0.83) in women by receiver operating characteristic (ROC) curve analysis [33]. Based on these and our studies [14, 15, 26, 27, 32–34], we believe that leptin may serve as an adipose tissue derived intermediate for the development of arterial stiffness, and a mediator in the pathophysiology of macrovascular diseases.

Reports have shown that several types of medications can affect arterial stiffness [35–40]. ACEi and ARB reduce central BP and augmentation index beyond the expected degree from the BP lowering effects, which is believed to be the result of reduction of oxidative stress and inflammation, and vasodilatation through angiotensin II inhibition [35, 36]. Treating older hypertensive patients with CCB resulted in a pronounced reduction of central aortic pressure and augmentation pressure as compared with placebo [35]. However, studies of β-blockers on arterial stiffness showed a lower effect on central BP decline compared with peripheral BP [36, 37]. The effects of statins on the reduction of aortic PWV are controversial, but recent study showed that a low dose of atorvastatin exerted beneficial effects on arterial stiffness and central aortic pressure in patients with mild HTN and hypercholesterolemia [38, 39]. Treatment with the peroxisome proliferator-activated receptor (PPAR) alpha agonist fenofibrate resulted in significant reduction in the augmentation index, PWV, and pro-inflammatory markers in obese patients without glucose intolerance [40]. Compared to these previous studies, our study showed that ACEi, ARB, β-blockers, CCB, statins, and fibrate had no influences on arterial stiffness in CAD patients.

The limitation of this study is that there was a lack of data regarding abdominal obesity to analyze the possible influence on arterial stiffness in our patients and it was a cross-sectional study with a limited number of CAD patients conducted at a single center. Therefore, the findings of this study should be confirmed by further longitudinal studies before a cause-effect relationship between serum leptin and arterial stiffness can be established in the CAD population.

## Conclusions

In conclusion, in this study which analyzing the role of leptin in angiographically diagnosed CAD patients, we found that serum leptin level correlated positively with cfPWV and is a predictor for the development of arterial stiffness. In addition, serum concentration of leptin correlated positively with the total number of stenotic coronary arteries in our CAD patients.

### Ethics approval and consent to participate

This study was approved by The Protection of the Human Subjects Institutional Review Board of Tzu-Chi University and Hospital. All participants gave written informed consent after thorough explanation of the procedures involved.

### Consent for publication

Not applicable.

### Availability of data and materials

All relevant data supporting the conclusions of this article is included within the article.

## Abbreviations

ACEi: angiotensin-converting enzyme inhibitors
ARB: angiotensin receptor blockers
BMI: body mass index
BUN: blood urea nitrogen
CAD: coronary artery disease
CCB: calcium channel blockers
cfPWV: carotid-femoral pulse wave velocity
Cre: creatinine
CVD: cardiovascular disease
DBP: diastolic blood pressure
DM: diabetes mellitus
EIA: enzyme immunoassay
ESH-ESC: European society of hypertension and the European society of cardiology
GFR: glomerular filtration rate
HDL-C: high-density lipoprotein cholesterol
HTN: hypertension
LDL-C: low-density lipoprotein cholesterol
MDRD: modification of diet in renal disease
SBP: systolic blood pressure
TCH: total cholesterol
TG: triglycerides
